# Radiotherapy alone for stage I-III low grade follicular lymphoma: long-term outcome and comparison of extended field and total nodal irradiation

**DOI:** 10.1186/1748-717X-7-103

**Published:** 2012-06-24

**Authors:** Matthias Guckenberger, Nikolaus Alexandrow, Michael Flentje

**Affiliations:** 1 Department of Radiation Oncology, University of Würzburg, Josef-Schneider-Str. 11, 97080, Würzburg, Germany

**Keywords:** Follicular lymphoma, Total nodal irradiation, Extended field irradiation

## Abstract

**Background:**

To analyze long-term results of radiotherapy alone for stage I-III low grade follicular lymphoma and to compare outcome after extended field irradiation (EFI) and total nodal irradiation (TNI).

**Methods and materials:**

Between 1982 and 2007, 107 patients were treated with radiotherapy alone for low grade follicular lymphoma at Ann Arbor stage I (n = 50), II (n = 36) and III (n = 21); 48 and 59 patients were treated with EFI and TNI, respectively. The median total dose in the first treatment series of the diaphragmatic side with larger lymphoma burden was 38 Gy (25 Gy – 50 Gy) and after an interval of median 30 days, a total dose of 28 Gy (12.6 Gy – 45 Gy) was given in the second treatment series completing TNI.

**Results:**

After a median follow-up of 14 years for living patients, 10-years and 15-years overall survival (OS) were 64% and 50%, respectively. Survival was not significantly different between stages I, II and III. TNI and EFI resulted in 15-years OS of 65% and 34% but patients treated with TNI were younger, had better performance status and higher stage of disease compared to patients treated with EFI. In multivariate analysis, only age at diagnosis (p < 0.001, relative risk [RR] 1.06) and Karnofsky performance status (p = 0.04, RR = 0.96) were significantly correlated with OS. Freedom from progression (FFP) was 58% and 56% after 10-years and 15-years, respectively. Recurrences outside the irradiated volume were significantly reduced after TNI compared to EFI; however, increased rates of in-field recurrences and extra-nodal out-of-field recurrence counterbalanced this effect resulting in no significant difference in FFP between TNI and EFI. In univariate analysis, FFP was significantly improved in stage I compared to stage II but no differences were observed between stages I/II and stage III. In multivariate analysis no patient or treatment parameter was correlated with FFP. Acute toxicity was significantly increased after TNI compared to EFI with a trend to increased late toxicity as well.

**Conclusions:**

Radiotherapy alone for stage I and II follicular lymphoma resulted in long-term OS with high rates of disease control; no benefit of TNI over EFI was observed. For stage III follicular lymphoma, TNI achieved promising OS and FFP and should be considered as a potentially curative treatment option.

## Background

Follicular lymphoma (FL) is after diffuse large B-cell lymphoma the second most common subtype of non-Hodgkin’s lymphomas in adults (~ 20–25%). Radiotherapy alone is an established curative treatment option for patients with stage I-II FL [1-3]. However, the volume, which needs to be irradiated, is still an issue of controversy. As the site of treatment failure is most frequently nodal outside irradiated involved volumes, extended-field irradiation (EFI) or even total nodal irradiation (TNI) have been proposed to improve outcome compared to involved-field irradiation (IFI). While studies reported improved progression free survival (PFS) after treatment of larger volumes, this did not transfer into improved overall survival (OS) [4,5]. Despite the majority of relapses are observed within 5 years after radiotherapy, late recurrences 15 – 20 years after treatment [4-6] indicate the need for studies with long-term follow-up, which are scarce in the literature.

Stage III disease is considered as non-curative and chemotherapy or wait and see strategies are most frequently perused. Nevertheless, studies reported promising disease control and overall survival rates after radiotherapy alone for stage III FL [7,8]. Recent data reported that lower radiation doses are sufficient for disease control [9], which might further reduce acute and especially long term toxicity. In this context of potentially reduced toxicity, radiotherapy in form of TNI might become a more attractive treatment strategy for advanced stage of disease. It was consequently the aim of this retrospective single institution study to describe long-term outcome after radiotherapy alone for stage I-III FL and to compare patterns of failure and OS after EFI and TNI.

## Material and methods

Between 1982 and 2007, 107 patients were treated with radiotherapy alone for low grade follicular lymphoma. Of these 107 patients, Ann Arbor stage was I (n = 50), II (n = 36) and III (n = 21). Ann Arbor stage was based on contrast enhanced whole body CT imaging and bone marrow biopsy in all patients.

All patients were treated with radiotherapy alone as primary treatment; patients who received chemotherapy as part of the primary treatment were excluded from this analysis. Patients were treated with either EFI of all central lymph node regions on the involved side of the diaphragm (supra-diaphragmatic: cervical, supra- and infraclavicular, mediastinal and hilar; infra-diaphragmatic: paraaortal, iliacal, inguinal) or with TNI (sequential supra-diaphragmatic and infra-diaphragmatic irradiation). Treatment was always started at the diaphragmatic side with higher lymphoma burden (first series) followed by the side with lower lymphoma burden or elective irradiation (second series). Total lymphatic irradiation with routine treatment of the Waldeyer’s tonsillar ring and mesenteric lymph nodes was not practiced. The spleen and Waldeyer's tonsillar ring were irradiated in 12% and 36% of the patients, respectively. Single fraction doses ranged between 1.5 Gy −2.0 Gy with the majority of patients treated with 1.8 Gy or 2.0 Gy. Lymph node regions without macroscopic involvement were treated with a total dose of 30 Gy and a boost of 6 - 8 Gy was given to macroscopic involved lymph node regions: the median total dose in the first treatment series was 38 Gy (25 Gy – 50 Gy) and after a median interval of 30 days, a total dose of 28 Gy (12.6 Gy – 45 Gy) was given in the second treatment series.

## Statistical analysis

Patient and treatment characteristics were compared between the two groups treated with EFI and TNI and between stage I/II and stage III using Chi-squared test for categorical variables and Mann-Withney *U*-test for continuous variables using Statistica X software (Statsoft, Tulsa, OK, USA). Survival and recurrence data were calculated by the Kaplan–Meier method: overall survival (OS) and freedom from progression (FFP) were calculated. Results between subgroups were compared using the log-rank test and the influence of patient and treatment factors on OS and FFP was evaluated using the multivariate Cox proportional-hazards regression model with backward exclusion of non-significant variables. Results with p < 0.05 were considered as statistically significant.

## Results

Patient characteristics and involved lymph node regions are summarized in Tables 1 and 2. Differences between EFI and TNI reflect the decision making process for the two approaches. Younger patients with better performance status were treated predominantly with TNI. Presence of B-symptoms, elevated serum LDH and bulky disease were not different between ENI and TNI. Additionally, no differences in total irradiation doses were observed.

**Table 1:** Patient and treatment characteristics with comparison between EFI and TNI and between stages I/II and stage III disease

	**All**		**EFI**		**TNI**		**p-value**	**Stage I/II**		**Stage III**		**p-value**
**# patients**	107		48		59			86		21		
**Age**							<0.001					<0.001
** median/range [years]**	57	26 - 83	66	37 - 83	51	26 - 74		61	27 – 83	51	26 - 68	
** ≥60 years**	48	45%	35	73%	13	22%		45	52%	3	14%	
**Sex [male]**	59	57%	26	55%	33	56%	NS	49	57%	10	48%	
**Karnofsky Performance status**							<0.001					NS
**median/range**	90	50 - 100	90	50 - 100	100	80 - 100		90	50 – 100	90	70 – 100	
**B-Symptoms**	1	1%	0	0%	1	2%	NS	0	0%	1	5%	NS
**LDH**							NS					0.03
** Elevated**	16	15%	9	19%	7	12%		8	9%	8	38%	
** Normal**	63	59%	29	60%	34	58%		55	64%	8	24%	
** unknown**	28	26%	10	21%	18	30%		23	27%	5	38%	
**Ann Arbor stage**							0.04					
** I**	50	47%	27	56%	23	39.0%						
** II**	36	34%	18	38%	18	30.5%						
** III**	21	20%	3	6%	18	30.5%						
**Bulk ≥7.5 cm**	5	5%	2	4%	3	5%	NS	3	3%	2	9%	NS
**Radiotherapy dose median/range [Gy]**	38	25 - 50	38	27 - 50	37	25 - 46	NS	37	25 - 50	37	29 - 46	NS

**Table 2:** Distribution of involved lymph node regions separately for patients with stage I, stage II and stage III follicular lymphoma

	**Neck**	**Axilla**	**Mediastinum**	**Paraaortal**	**Iliakal**	**Inguinal**	**Unknown**
**Stage I (n = 50)**	46%	8%	0%	0%	2%	32%	12%
**Stage II (n = 36)**	81%	39%	31%	36%	36%	56%	3%
**Stag III (n = 21)**	76%	62%	24%	67%	29%	48%	0%

The median number of involved lymph node regions was 2 (range 2–5) and 4 (range 2–11) in stage II and III disease, respectively. Patients at stage III were younger compared to stage I/II and elevated LDH was observed more frequently in stage III patients; there were no difference in the rate of bulky disease and irradiation doses. Of patients with stage III disease, 18/21 were treated with TNI; one patient denied infra-diaphragmatic after supra-diaphragmatic irradiation and the reason for two patients is unknown.

Median follow-up was 10 years for all patients (0.3 – 26 years) and 14 years (2–26 years) for living patients. Follow-up was not different between EFI and TNI and between stages I/II and stage III.

**Figure 1:**
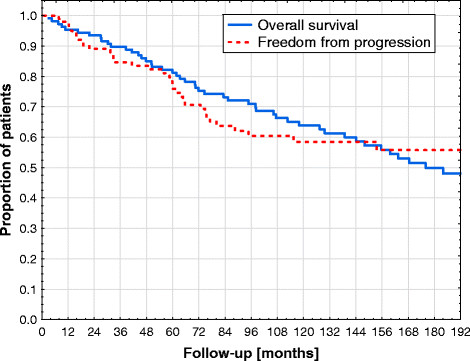
Kaplan Meier Curves showing overall survival (OS) and freedom from disease progression (FFP) for all patients.

**Figure 2:**
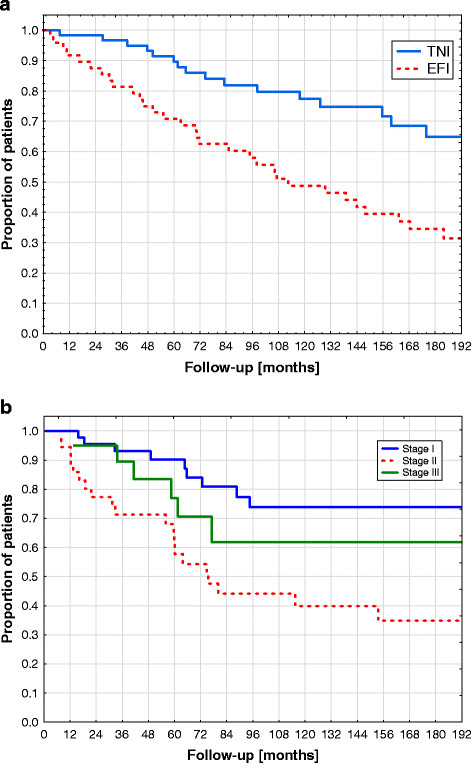
**Kaplan Meier Curves showing overall survival in relationship to TNI versus EFI (no significant difference) (Figure** 2**a) and stage of disease (no significant differences) (Figure** 2**b)**

For the total patient population, 10-years and 15-years OS were 64% and 50% (Figure 1), respectively. In univariate analysis, age > median of 60 years (p = 0.0001), EFI versus TNI (p = 0.001) and Karnofsky performance status <100 (p = 0.0003) were correlated with decreased OS. Median age of 60 years differentiated between 15-years OS of 67% and 29%; TNI and EFI resulted in 15-years OS of 65% and 34% (Figure 2a), respectively. Overall survival was not significantly different between stages I-III: 15-years OS were 51%, 45% and 54% for patients with stage I, II and III (Figure 2b), respectively. In Cox proportional-hazards analysis (with age, TNI vs ENI, Karnofsky performance status and Ann Arbor stage included into the modelling), age at diagnosis (p < 0.001 and relative risk of 1.06) and Karnofsky performance status (p = 0.04 and relative risk of 0.96) were significantly correlated with OS (Table 3).

**Table 3:** Univariate and multivariate analysis of patient and treatment factors influencing overall survival and freedom from disease progression

	**Overall Survival**			**Freedom from disease progression**		
	**Univariate analysis**	**Multivariate analysis**		**Univariate analysis**	**Multivariate analysis**	
**Age**	p = 0.0001	p = 0.0001	RR = 1.06	NS		
**Karnofsky performance status**	p = 0.0003	P = 0.04	RR = 0.96	NS		
**Stage of disease**	NS			P = 0.001	NS	NS
**TNI vs EFI**	p = 0.001	NS		NS		

**Figure 3:**
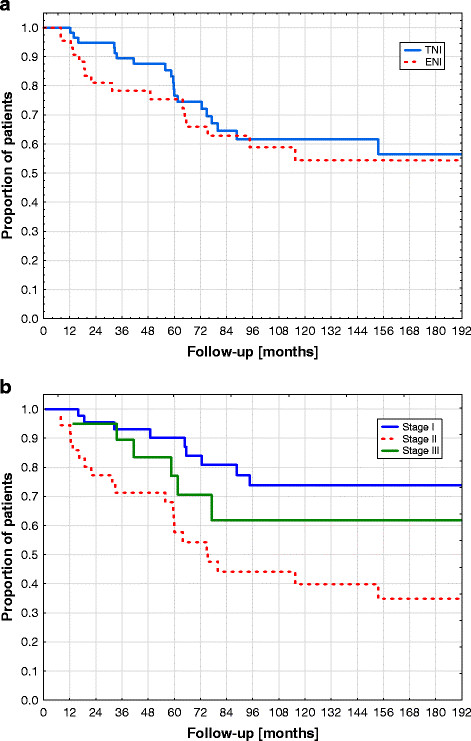
**Kaplan Meier Curves showing freedom from disease progression in relationship to TNI versus EFI (no significant difference) (Figure** 2**a) and stage of disease (significant difference between stage I and stage II) (Figure** 2**b)**

FFP was 76%, 58% and 56% after 5-years, 10-years and 15-years (Figure 1), respectively. Only one recurrence was observed after 10 year (12.8 years). FFP at 10 years was not different between TNI and EFI with 62% and 54% (p = 0.50) (Figure 3a), respectively. FFP at 10 years was 74%, 40% and 62% for stage I, stage II and stage III (Figure 3b), respectively, and results of stage II were significantly worse compared to stage I (p = 0.001). In Cox proportional-hazards analysis (with Ann Arbor stage, age and TNI vs ENI included into the modelling), no patient or treatment characteristic was correlated with FFP.

A total of 35 recurrences were observed with crude rates of 33% and 32% after EFI and TNI, respectively. 14/16 (87.5%) and 8/19 (42%) of the recurrences after EFI and TNI were located outside the irradiated volume, which was highly significant (p = 0.01).

During the first irradiation series, 5 patients (5%) developed acute toxicity grade III (gastrointestinal mucositis n = 4; nausea and vomiting n = 1). During the second irradiation series in TNI patients, acute toxicity was significantly increased with 1 patient (2%) developing grade IV toxicity (thrombocytopenia) and 8 patients (14%) suffering from grade III toxicity (leukopenia n = 3, thrombocytopenia n = 2, gastrointestinal mucositis n = 2, nausea and vomiting n = 1).

Late toxicity was maximum grade II in all patients and grade II toxicity was observed in 27 patients (25%). The most frequent grade II toxicity was pulmonary (n = 12; pulmonary fibrosis, dyspnoea, cough) and gastro-intestinal (n = 9; dry mouth, dysphagia, dental, hepatitis, colitis). Two patients developed chronic haematological toxicity and one patient generalized dry skin and fatigue. Any grade II toxicity was increased after TNI (n = 18; 31%) compared to EFI (n = 8; 17%) but this difference did not reach statistical significance.

## Discussion

This study confirms previous data, that treatment of larger irradiation fields does not improve overall survival in stage I and II follicular lymphoma. Wilder et al. reported the M.D. Anderson experience of 80 patients treated with IFI (9%), regional RT (54%; treatment of 1–3 adjacent, grossly uninvolved nodal regions) or EFI (37%); TNI irradiation was not practiced [5]. After a median follow-up of 19 years for living patients, no difference in OS was observed between EFI and IFI/regional RT: 49% versus 40% at 15 years. Recurrences were more frequently observed in patients treated with EFI; however, there were imbalances of stage distribution and total irradiation dose and no multivariate analysis was performed. Mac Manus et al. described the Stanford experience of 177 patients and differentiated between irradiation of the involved side or both sides of the diaphragm [4]. After a median follow-up of 7.7 years, FFP at 10 year were 36% and 67% for treatment of the involved side only and both sides of the diaphragm, respectively, which was highly significant. However, this improvement in disease control did not transfer into increased OS.

In our own study, evaluating the impact of TNI and EFI on FFP and OS was difficult because of a correlation between the target volume concept and established prognostic parameters: age of the patients, performance status and stage of disease. No difference of FFP was observed between TNI and EFI in univariate and multivariate analysis. In agreement to Stuschke et al. [10], irradiation of uninvolved lymph node regions with a median dose of 30 Gy was effective in reducing out-of-field recurrences. However, an increased rate of in-field recurrences and extra-nodal out-of-field recurrence counterbalanced this beneficial effect of TNI. Regarding OS, patients treated with TNI were younger and had a better performance status and as a consequence, 15-years OS was 65% and 34% after TNI and EFI, respectively. However, only age and Karnofsky performance status and not the target volume concept remained statistically significant in multivariate analysis.

The 10-years OS of 64% for the patient population with stages I/II disease is considered as representative for early stage follicular lymphoma because our department is the only radiotherapy centre within a radius of minimum 50 – 100 km and rather strict referral patters in the geographical region; a potential patient selection bias is consequently limited. The observed OS is in good agreement with published data in the literature about primary radiotherapy for stages I/II follicular lymphoma: two recent literature reviews reported 10-years OS rates ranging between 46% and 79% (weighted average of 63% in Heinzelmann et al.) after primary radiotherapy using mostly IFI or EFI [11,12]. No obvious correlation of OS and irradiated volume is seen in this literature review. This further supports the hypothesis of no OS benefit after TNI.

The larger treatment volumes of TNI came at the price of increased toxicity. While the first series of EFI irradiation was well tolerated with only 5% of the patients developing grade III toxicity, 16% of the patients suffered from acute toxicity grade ≥ III during the second series, which completed the TNI. Additionally, chronic toxicity grade II was observed more frequently after TNI (31%) compared to EFI (17%).

Randomized data about the question of the appropriate irradiation volume are missing. A German multicentre phase III trial, which randomized between TNI and EFI, closed in 2007 and final results are awaited. Until results of this trial are available, increased acute and chronic toxicity with no apparent benefit in terms of survival and disease control as observed in this study do not support the concept of TNI compared to EFI for stage I and II FL.

Twenty one patients with stage III FL were treated with radiotherapy alone and were included into this study. Unfortunately, prognostic factors were not fully balanced between stages I/II and stage III. Patients at stage III FL were younger indicating that age was a selection criterion for radiotherapy alone in stage III patients. As expected based on the increased lymphoma burden, LDH was more frequently elevated in stage III patients. TNI was the planned treatment in all stage III patients and was successfully completed in almost 90% of the patients.

Outcome was favourable with 10-years OS and FFP of 61% and 62%, respectively. OS and FFP were not significantly different to stages I/II. This favourable outcome is in good agreement with data in the literature [7,8,13,14], where 10-years OS ranged between 47% and 65%. Only a small number of patients with stage III disease was at risk after 10 years in our study; however, the Stanford experience of 66 stage III patients treated with radiotherapy alone described no disease recurrence after 10 years and only 5/29 lymphoma deaths after 10 years [8]. These data are promising especially in the light of the recently published British trial, which showed that radiotherapy doses for indolent lymphomas can be reduced without negatively affecting clinical outcome. Consequently, TNI with reduced irradiation may have the potential of long term disease control and survival with simultaneously decreased acute and chronic toxicity [9]. Of course, such concepts need to be validated within clinical trials.

Some limitations of our study need to be discussed. The retrospective nature of this study is associated with well-known limitations. The decision making process between EFI and TNI considered well established prognostic factors like age, performance status and stage of FL, which made the statistical comparison of the two target volume concepts difficult. Additionally, the reconstruction of the decision making process in favour of radiotherapy and against chemotherapy in stage III FL could not be fully reconstructed: the ratio of stage I + II : stage III patients in the International Lymphoma classification project was 2:1 [15] compared to 4:1 in our study, which suggests some selection process for radiotherapy.

## Conclusions

Radiotherapy alone for stage I and II follicular lymphoma was an effective and curative treatment with high rates of disease control. Larger irradiation fields covering both sides of the diaphragm did not improve tumor control and overall survival. Additionally, TNI was associated with increased rates of acute and chronic toxicity. Based on this study and data from the literature, EFI or IFI are the preferred treatments for stage I and II follicular lymphoma. In patients with stage III FL, TNI resulted in promising tumor control and overall survival and should be considered as a potentially curative treatment option.

## Competing interests

The authors declare that they have no competing interest.

## Authors’ contributions

MG designed the study, participated in data collection and performed the data analysis. NA collected the data and participated in the data analysis. MF participated in the design of the study. All authors performed critical review of the manuscript and finally approved the manuscript.
